# Patterns of Non‐influenza Respiratory Viruses Among Severe Acute Respiratory Infection Cases in Burkina Faso: A Surveillance Study

**DOI:** 10.1111/irv.13271

**Published:** 2024-03-19

**Authors:** Moussa Lingani, Assana Cissé, Abdoul Kader Ilboudo, Issaka Yaméogo, Zekiba Tarnagada

**Affiliations:** ^1^ Laboratoire National de Référence‐Grippes Institut de Recherche en Sciences de la Santé (LNRG‐IRSS) Ouagadougou Burkina Faso; ^2^ Unité de Recherche Clinique de Nanoro Institut de Recherche en Sciences de la Santé (IRSS‐URCN) Nanoro Burkina Faso; ^3^ One Health Association Burkina Faso Ouagadougou Burkina Faso; ^4^ Service de surveillance épidémiologique Ministère de la santé et de l'Hygiène publique Ouagadougou Burkina Faso

**Keywords:** Burkina Faso, etiologies, non‐influenza virus, respiratory tract infections

## Abstract

**Background:**

Although influenza viruses cause only one‐fifth of severe acute respiratory infections (SARI) in Burkina Faso, the other viral causes of SARI remain poorly investigated to inform clinical and preventive decision making.

**Methods:**

Between 2016 and 2019, we prospectively enrolled inpatients meeting the World Health Organization (WHO) case definition of SARI in Burkina Faso. Results of viral etiologies among inpatients tested negative for influenza using the Fast Track Diagnostics Respiratory Kits (FTD‐33) were reported.

**Results:**

Of 1541 specimens tested, at least one respiratory virus was detected in 76.1% of the 1231 specimens negative for influenza virus. Human rhinoviruses (hRVs) were the most detected pathogens (476; 38.7%), followed by human adenoviruses (hAdV) (17.1%, 210/1231), human respiratory syncytial virus (hRSV) (15.4%, 189/1231), enterovirus (EnV) (11.2%, 138/1231), human bocavirus (hBoV) (7.9%, 97/1231), parainfluenza 3 (hPIV3) (6.1%, 75/1231), human metapneumovirus (hMPV) (6.0%,74/1321), parainfluenza 4 (hPIV4) (4.1%, 51/1231), human coronavirus OC43 (hCoV‐OC43) (3.4%, 42/1231), human coronavirus HKU1(hCoV‐HKU1) (2.7%, 33/1231), human coronavirus NL63 (hCoV‐NL63) (2.5%, 31/1231), parainfluenza 1 (hPIV1) (2.0%, 25/1231), parainfluenza 2 (hPIV2) (1.8%, 22/1231), human parechovirus (PeV) (1.1%, 14/1231), and human coronavirus 229E (hCoV‐229E) (0.9%, 11/1231). Among SARI cases, infants aged 1–4 years were mostly affected (50.7%; 622/1231), followed by those <1 year of age (35.7%; 438/1231). Most detected pathogens had year‐long circulation patterns, with seasonal peaks mainly observed during the cold and dry seasons.

**Conclusion:**

Several non‐influenza viruses are cause of SARI in Burkina Faso. The integration of the most common pathogens into the routine influenza surveillance system might be beneficial.

**Abbreviations**
AU MS: African union member states
EnV: enterovirus
hAdV: human adenoviruses
hBoV: human bocavirus
hCoV‐229E: human coronavirus 229E
hCoV‐HKU1: human coronavirus HKU1
hCoV‐NL63: human coronavirus NL63
hCoV‐OC43: human coronavirus OC43
hMPV: human metapneumovirus
hPIV 1–4: parainfluenza 1–4
hRSV: human respiratory syncytial virus
hRVs: human rhinoviruses
ILI: influenza‐like illness
NIRL: National Influenza Reference Laboratory
PeV: parechovirus
RT‐PCR: reverse‐transcription polymerase chain reaction
SARI: severe acute respiratory infection
WHO: World Health Organization

## 1 Background

Severe acute respiratory infections (SARI) are a real public health problem worldwide due to their high morbidity and mortality [1]. At a global level, lower respiratory infections remain the world's most deadly communicable disease, ranked as the fourth leading cause of death and second leading cause of death in low‐income countries [2]. In 2019, nearly 2.6 million deaths were recorded at global level, among them 400,000 were from the Western sub‐Saharan Africa [3].

Most deaths in sub‐Saharan Africa are indirectly attributable to delayed or inadequate care because of limited laboratory diagnosis capacities [4, 5]. Indeed, access to reliable diagnostic testing is severely limited in this region, and misdiagnosis commonly occurs [5]. Understandably, the allocation of resources to diagnostic laboratory testing has not been a priority for resource‐limited health care systems, but unreliable and inaccurate laboratory diagnostic testing leads to unnecessary expenditures in a region already plagued by resource shortages [6].

Viral infections are major causes of SARI in sub‐Saharan Africa, but the responsibility of viruses other than influenza remains poorly documented despite that influenza causes only 20% of these infections [7, 8, 9, 10]. A study in China showed that in addition to influenza A and B viruses, other viruses such as the human respiratory syncytial viruses (hRSV), enteroviruses (EnV), human metapneumovirus (hMPV), rhinovirus (hRV), adenovirus, and parainfluenza virus (hPIV) types 1–4 were also commonly causing severe respiratory infections [11]. In sub‐Saharan Africa however, most studies were limited to influenza viruses [12, 13, 14] and severe acute respiratory syndrome coronavirus 2 (SARS‐CoV‐2) [15], overlooking the other viral causes of SARI despite that most inpatients were negative for influenza [8, 9, 10, 16]. The presence of viral pathogens in severe clinical forms of common respiratory conditions such bronchiolitis, pneumonia, laryngitis, and asthma is reported [17]; however, the etiologic pathogens are not investigated in practice due to an underestimation of their role in SARI because of severe limitations in laboratory diagnosis capacities and treatment options as in developed countries [18, 19, 20]. The adequate control of these infections in developed countries is mainly based on a better understanding of the epidemiology and the availability of adequate laboratory diagnosis capacity.

In Burkina Faso, a sentinel surveillance system for influenza viruses was established in 2010, which has helped to identify and characterize circulating seasonal influenza A(H3N2), A(H1N1) pdm09, and influenza B viruses [8, 9, 10]. This surveillance system has contributed to identify high‐risk groups in need of targeted interventions [8, 9, 10]. Given the limited laboratory resources in the country, the diagnosis of other respiratory viruses has not been included among the priorities and was usually retrospective and limited to children under 5 years old in university teaching hospitals [21, 22]. The studied pathogens were also limited to the hRSV, metapneumovirus (hMPV), adenovirus (hAdV), parainfluenza viruses 1, 2, and 3 (hPIV 1–4), and rhinovirus/enterovirus (hRVs) [21, 22]. This study aimed to describe the epidemiological and virological patterns of SARI cases tested negative for influenza infections in Burkina Faso using the platform of the national influenza surveillance of the country.

## 2 Material and Methods

### 2.1 Study Sites and Data Collection

Influenza surveillance was conducted among inpatients of all ages at the Bogodogo University Teaching Hospital in Ouagadougou, the capital city, and in three district hospitals including that of Boussé in the “Plateau Central” health region, that of Kongoussi in the “Centre Nord” health region, and that of Houndé in the “Hauts Bassins” health region. SARI cases were identified using the World Health Organization case definition (hospitalized patient with an acute respiratory infection, with a history of fever or measured temperature ≥38°C and cough, with onset within the last 10 days, and requiring hospitalization). Trained health workers identified cases 7 days a week throughout the study period and completed a paper‐based case report form for each patient. Collected information included the socio‐demographic characteristics, clinical symptoms, prior treatments, and hospitalization outcomes. In addition, for each patient, nasopharyngeal (NP) and oropharyngeal (OP) specimens were collected within 24 h of hospitalization and placed into a tube of Universal Transport Media (Copan Diagnostics). Specimens were immediately refrigerated at 2–8°C for temporary storage and subsequently shipped to the NIRL within 72 h after collection, aliquoted for testing or long‐term storage in minus 80°C freezers [7].

### 2.2 Laboratory Testing Methods

#### 2.2.1 Nucleic Acid Extraction

The detailed laboratory work was described elsewhere [7]. Briefly, total nucleic acid (TNA) was extracted from 400 μL of Universal Transport Media that contained the specimens and was eluted into 110 μL using the EZ1 Advanced XL Instrument with the EZ1 Mini Viral 2.0 Kit (Qiagen) following the manufacturer's instructions. The extracts were screened for the detection of respiratory pathogens using the Fast Track Diagnostics 33 (FTD‐33) reverse‐transcriptase real‐time polymerase chain reactions (rRT‐PCR) kits [7, 23]. The kits were used for the detection of the following respiratory viruses, bacteria, and fungi: *influenza A*, *influenza A* subtype A(H1N1)pdm09, *influenza B*, and *influenza C*; parainfluenza viruses 1, 2, 3, and 4; coronaviruses NL63, 229E, OC43, and HKU1; *human metapneumoviruses* A and B; rhinovirus; *respiratory syncytial viruses* A and B; adenovirus; enterovirus; parechovirus; bocavirus; *Pneumocystis jirovecii*; *Mycoplasma pneumoniae* ; *Chlamydia pneumoniae* ; *Streptococcus pneumoniae* ; *Haemophilus influenzae* ; *H. influenzae* type B; *Staphylococcus aureus* ; *Moraxella catarrhalis* ; *Bordetella* species (excluding *Bordetella parapertussis* ); *Klebsiella pneumoniae* ; *Legionella* species; and *Salmonella* species following the manufacturer's instructions [24]. All assays were conducted using the Applied Biosystems 7500 Real‐Time PCR Instrument (Thermo Fisher Scientific) [7].

### 2.3 Data Analysis

Statistical analysis was conducted using R, RStudio, and the Tidyverse package. Age groups <1 years, 1–4 years, 5–18 years, and 19+ years were considered. Descriptive analyses comprised assessing frequency distributions and proportions for each variable category. Graphs were plotted using Excel. Fisher's exact test for categorical variables were used for group comparisons. For result interpretation, *p*‐values <0.05 were considered statistically significant.

## 3 Results

Over the study period, 1541 SARI specimens were collected. Of them, 310 (20.1%) were tested positive for influenza viruses. This report focused on the 1231 patients tested negative for influenza. Most patients were male (56.4%, 694/1231) and infants between 1 and 4 years of age were mainly affected (50.7%, 622/1228), followed by infants below 1 year of age (35.7%, 438/1228). Most patients came from rural areas (57.4%, 707/1231) (Table 1).

**TABLE 1:** General characteristics of the study population, 2016–2019, in Burkina Faso.

Characteristics		Total numbers	Percentage
Sex			
	Female	537	43.6
	Male	694	56.4
Residency			
	Urban	524	42.6
	Rural	707	57.4
Year of sampling			
	2016	32	2.6
	2017	514	41.8
	2018	580	47.1
	2019	105	8.5
Age group (in years)			
	<1	438	35.7
	1–4	622	50.7
	5–18	75	6.1
	19+	93	7.6
	Missing^a^	3	—
Health center			
	Bogodogo	298	24.2
	Houndé	377	30.6
	Kongoussi	362	29.4
	Boussé	194	15.8
Outcome			
	Cured	931	81.2
	Death	22	1.9
	In treatment	194	16.9
	Missing^a^	84	—

^a^ Missing values were not included in the calculation of proportions.

### 3.1 Viral Pathogens Detected

Among the 1231 samples tested, 937 (76.1%) were positive for at least one of the pathogens tested. The most represented pathogen was the rhinovirus (38.7%), followed by adenovirus (17.1%) and respiratory syncytial virus (in 15.4%). Parainfluenza viruses were detected in 13.6% of the sample, while coronaviruses were detected in nearly 10% of tested specimens (Table 2). The proportions of bocaviruses and rhinoviruses infections were significantly higher in rural area than urban areas (*p* < 0.05) (Table 3).

**TABLE 2:** Proportions of detected other viral pathogens among SARI cases in Burkina Faso, 2016–2019 (*n* = 1231).

Detected pathogens	Subtype (if any)	Positive cases (*n*)	Positive cases (%)
At least one pathogen detected		937	76.1
Adenovirus		210	17.1
Bocavirus		97	7.9
At least one coronavirus type		113	9.2
	*Coronavirus229Ee*	11	0.9
	*CoronavirusHKU1*	33	2.7
	*CoronavirusNL63*	31	2.5
	*CoronavirusOC43*	42	3.4
Enterovirus		138	11.2
Human metapneumovirus A and B		74	6.0
Parainfluenza virus		167	13.6
	*Human parainfluenza 1*	25	2.0
	*Human parainfluenza 2*	22	1.8
	*Human parainfluenza 3*	75	6.1
	*Human parainfluenza 4*	51	4.1
Parechovirus		14	1.1
Respiratory syncytial viruses A and B		189	15.4
Rhinovirus		476	38.7

Abbreviation: SARI, severe acute respiratory infection.

**TABLE 3:** Other respiratory viruses among severe acute severe respiratory infection patients according to sociodemographic characteristics 2016–2019, Burkina Faso (*n* = 1231).

Detected pathogens	Age groups in years (*n*, %)					Settings (*n*, %)			Sex (*n*, %)		
	<1	1–4	5–18	19+	*p*‐value (χ2)	Urban	Rural	*p*‐value (χ2)	Female	Male	*p*‐value (χ2)
At least one pathogen	372 (30.3)	490 (39.9)	40 (3.3)	34 (2.8)	<0.001	419 (34.0)	518 (42.1)	<0.01	387 (31.4)	550 (44.7)	<0.01
Adenovirus	72 (5.9)	122 (9.9)	11 (0.9)	5 (0.4)	<0.01	99 (8.0)	111 (9.0)	0.15	80 (6.5)	130 (10.6)	0.08
Bocavirus	32 (2.6)	60 (4.9)	3 (0.2)	2 (0.2)	0.03	39 (3.2)	58 (4.7)	0.67	31 (2.5)	66 (5.4)	0.02
All coronaviruses	46 (3.7)	57 (4.6)	5 (0.4)	5 (0.4)	0.43	37 (3.0)	76 (6.2)	0.03	45 (3.7)	68 (5.5)	0.42
Coronavirus229e	1 (0.1)	10 (0.8)	0 (0.0)	0 (0.0)	0.11	2 (0.2)	9 (0.7)	0.13	3 (0.2)	8 (0.6)	0.37
Coronavirushku1	16 (1.3)	15 (1.2)	0 (0.0)	2 (0.2)	0.32	6 (0.5)	27 (2.2)	<0.01	15 (1.2)	18 (1.5)	0.86
Coronavirusnl63	15 (1.2)	15 (1.2)	1 (0.1)	0 (0.0)	0.26	18 (1.5)	13 (1.1)	0.10	12 (1.0)	19 (1.5)	0.71
Coronavirusoc43	16 (1.3)	19 (1.5)	4 (0.3)	3 (0.2)	0.67	13 (1.1)	29 (2.4)	0.15	18 (1.5)	24 (1.9)	0.99
Enterovirus	56 (4.6)	79 (6.4)	3 (0.2)	0 (0.0)	<0.001	61 (5.0)	77 (6.3)	0.72	52 (4.2)	86 (7.0)	0.15
Metapneumoviruses	29 (2.4)	40 (3.3)	4 (0.3)	1 (0.1)	0.16	27 (2.2)	47 (3.8)	0.33	36 (2.9)	38 (3.1)	0.40
All parainfluenza virus	59 (4.8)	94 (7.7)	7 (0.6)	6 (0.5)	0.09	93 (7.6)	74 (6.0)	<0.001	65 (5.3)	102 (8.3)	0.21
Parainfluenza 1	4 (0.3)	20 (1.6)	1 (0.1)	0 (0.0)	0.03	16 (1.3)	9 (0.7)	0.04	9 (0.7)	16 (1.3)	0.54
Parainfluenza 2	8 (0.7)	12 (1.0)	1 (0.1)	1 (0.1)	0.99	15 (1.2)	7 (0.6)	0.02	7 (0.6)	15 (1.2)	0.29
Parainfluenza 3	36 (2.9)	32 (2.6)	4 (0.3)	4 (0.2)	0.08	42 (3.4)	33 (2.7)	0.02	33 (2.7)	42 (3.4)	0.99
Parainfluenza 4	13 (1.1)	33 (2.7)	2 (0.2)	3 (0.2)	0.29	23 (1.9)	28 (2.3)	0.77	19 (1.5)	32 (2.6)	0.39
Parechovirus	10 (0.8)	3 (0.2)	1 (0.1)	0 (0.0)	0.04	7 (0.6)	7 (0.6)	0.60	3 (0.2)	11 (0.9)	0.11
Respiratory syncytial viruses	113 (9.2)	64 (5.1)	9 (0.7)	3 (0.2)	<0.001	106 (8.6)	83 (6.7)	<0.001	77 (6.3)	112 (9.1)	0.43
Rhinovirus	183 (14.9)	256 (20.8)	21 (1.7)	16 (1.3)	<0.001	204 (16.6)	272 (22.1)	0.91	193 (15.7)	283 (23.0)	0.09

*Note:* Each pathogen was screened in all participants (1231); therefore, overlapping numbers are reported.

Abbreviation: *n*, number of positive cases.

**FIGURE 1:**
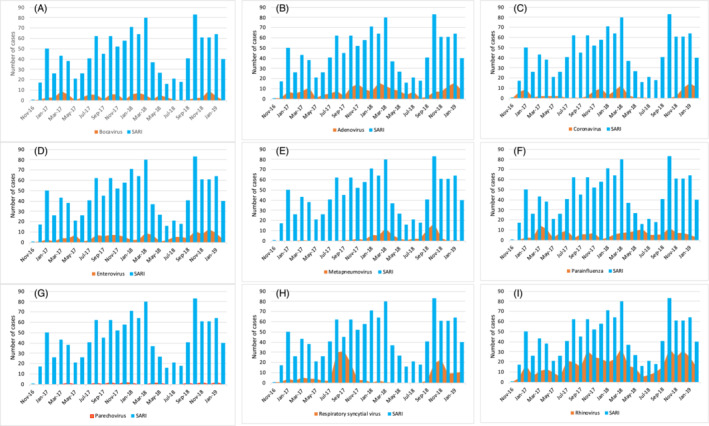
Monthly distribution of severe acute respiratory infection cases and confirmed other respiratory viruses from 2016 to 2019, Burkina Faso. The figure shows the monthly trend of SARI cases and the contribution of each pathogen or group of pathogens among the cases. SARI, severe acute respiratory infection.

Transmission greatly varied according to the seasons and the type of pathogens tested (Figure 1). For the respiratory syncytial viruses, two peak transmissions were observed towards the end of the rainy season and covered the cold and dry seasons (Figure 1H). A year‐long transmission was reported for rhinovirus (Figure 1I), for adenoviruses (Figure 1B), enteroviruses (Figure 1D), and parainfluenza viruses (Figure 1F). For coronaviruses, year‐long transmissions were reported with peak transmission during the cold and dry seasons (Figure 1C).

## 4 Discussion

This is the first report of other respiratory viruses among SARI cases tested negative for influenza infections in Burkina Faso with the aim of describing the contribution of other respiratory pathogens in the high burden of disease in the country.

The overall pathogen detection rate was nearly 75% among SARI patients. Our finding was consistent with results from developed countries where more accurate data are commonly available [25, 26, 27]. Indeed, in a recent study from Japan, nearly 69% of tested patients were positive for at least one viral pathogen other than influenza infections [18], and prevalence was nearly 50% in South Korea [27] and 91% in a study from China [25]. In developing countries, similar trends were observed in Niger a country neighboring the current study setting with similar climatic patterns and where the reported prevalence was 86% [28]. The overall prevalence however varied according to the age groups, and in the current study, children below 12 months of age and preschool‐age children 1–4 years of age were the most affected. This trend was also supported by a study in Cameron among adults, which reported a low prevalence of 25% [29]. A study conducted among children below 5 years of age reported much higher prevalence with nearly 83% although this included influenza infections [30]. All those studies reported a high prevalence of other respiratory viruses with similar finding across developed and developing countries and suggest that control measures are needed in all setting even where prevalence data are not fully documented.

Rhinoviruses were the most frequently detected pathogens similar to that reported in other regions. Indeed, the prevalence in the current study was 38% similar to that of India, which was 42% [31]. Lower trends were however reported in Madagascar, where rhinoviruses represented the most frequently detected pathogens but in lower proportion (24.8%) [32]. In Cote d'Ivoire, a neighboring country of Burkina Faso, rhinovirus also represented the most common pathogens detected and suggests that a similar trend may exist across the West African region calling for regional policies [33]. The temporal distribution did not however follow specific seasonal patterns although peak transmissions were often observed. This suggests that rhinoviruses should be the first pathogens to be investigated in SARI patients tested negative for influenza infections in Burkina Faso. Children were particularly affected as describe elsewhere, and studies suggest that the infection rate was highest among infant age 6 months to 2 years [34]. As rhinoviruses frequently cause severe infections in young children, their management might be indicated in SARI children tested negative for influenza infection and where rhinovirus diagnostic is unavailable.

Adenoviruses were the second most commonly detected pathogens particularly among the under 5‐years‐old children. Similar trend was reported in China, and 15.85% of patients were infected with adenovirus [35]. In other settings, adenovirus were less common with 4% of patients infected [36]. These differences suggest that there is needed for continuous surveillance in each setting as geographic variations can direct control strategies. Seasonal trends were observed, and the peak transmission commonly starts from the end of the rainy season and covers the cold and dry periods [37]. Seasonality should therefore be a key parameter during the planning of control interventions.

Respiratory syncytial viruses were the third most commonly detected pathogens and was prevalent among children aged below 4 years. In an Indian study, although the prevalence of RSV was similar to that reported within the current study (20%), the infection was ranked as the second most commonly diagnosed condition [31]. The virus was detected year‐round, and peak transmission periods were observed during the cold and dry seasons. In a study conducted in a neighboring country, RSV was however the most commonly detected infection with a prevalence of 31.92% but that study was solely conducted during the cold and dry seasons coinciding with the peak transmission period [33].

Parainfluenza viruses were the fourth commonly detected pathogens. Children below 4 years of age remained the most affected populations and transmission occurred year long. As parainfluenza infections represent important causes of SARI, targeted interventions are thus needed to reduce its burden [38, 39]. Our finding was similar to that reported in Ivory Coast where the prevalence of the pathogen was 20% [33] and in Niger with 24% [28]. Regional strategies could be useful to standardized interventions.

Enteroviruses were the fifth most commonly detected pathogens with peak transmission during the cold and dry seasons. In a study conducted in Ivory Coast, the prevalence of enteroviruses was less than 1% [33]. The climatic differences between the two countries may be behind these variations although the countries share common borders [40]. Indeed, Burkina Faso is in the majority, a Sahelian country where the Harmattan wind is common and associated with circulation of several virus [33, 40, 41].

The coronaviruses detection rate was 9% with peak transmissions during the cold and dry seasons. Similar trend was observed in hospitalized children in Slovenia with 6% of infection [42]. Some studies have found low rates of detection of coronaviruses [18]. Studies have also reported that the prevalence of these coronavirus in pediatric patients can be significantly higher, varying from about 5% to more than 30%, and these discrepancies could be attributed to differences in the research methodologies. In the Japan study, no case of HCoV‐OC43 and HCoV‐229E nor HCoV‐NL63 and HCoV‐HKU1 were detected, and this discrepancy can be explained by the seasonality of these infections [18].

Metapneumoviruses were also detected in a considerable proportions (6%) with transmission occurring mostly during the dry and cold seasons of the year. The prevalence was similar to that reported in a study conducted in the same region [33]. Previous studies have reported that hMPV was prevalent during late winter and spring in temperate climates [43, 44], but in our study, metapneumovirus was detected year‐long although peak transmission period coincided with pattern already described by other studies [43, 44].

This study has some limitations as the pathogens tested were limited to that of the FTD‐33 diagnostic capacity, thus leaving other pathogens unaddressed. However, this already documents most commonly pathogens involved in SARI in the country and might be useful for the health system.

## 5 Conclusion

This report showed that other respiratory viruses play an important role in the etiology of SARI mainly among children under 4 years of age. Therefore, it would be beneficial to extend surveillance to these pathogens for a better understanding of their epidemiology.

## Author Contributions

**Moussa Lingani:** Data curation; Formal analysis; Software; Validation; Visualization; Writing – original draft. **Assana Cissé:** Investigation; Methodology; Project administration; Resources; Supervision; Writing – review and editing. **Abdoul Kader Ilboudo:** Data curation; Investigation; Supervision; Validation; Writing – review and editing. **Issaka Yaméogo:** Investigation; Methodology; Writing – review and editing. **Zekiba Tarnagada:** Conceptualization; Funding acquisition; Investigation; Methodology; Project administration; Resources; Supervision; Validation; Writing – review and editing.

## Ethics Statement

The Burkina Faso influenza surveillance is a national program approved by the Ministry of Health and coordinated by the National Influenza Reference Laboratory (NIRL) as part of influenza pandemic preparedness. All patients who participated in this surveillance provided however provided informed consent in accordance with Helsinki recommendation.

## Conflicts of Interest

The authors declare no conflicts of interest.

### Peer Review

The peer review history for this article is available at https://www.webofscience.com/api/gateway/wos/peer‐review/10.1111/irv.13271.

## Data Availability

The data that support the findings of this study are available from the corresponding author upon reasonable request.
